# Patients’ satisfaction towards radiological service and associated factors in Hawassa University Teaching and referral hospital, Southern Ethiopia

**DOI:** 10.1186/s12913-017-2384-z

**Published:** 2017-06-26

**Authors:** Teshome Mulisa, Fasil Tessema, Hailu Merga

**Affiliations:** 1 Department of Radiology, Hawassa University Teaching and Referral Hospital, Hawassa, Ethiopia; 2 Department of Epidemiology, College of Health Sciences, Jimma University, Jimma, Ethiopia

**Keywords:** Patient’s satisfaction, Radiological services, Associated factors, Ethiopia

## Abstract

**Background:**

Patient satisfaction, one of the main components of quality of care, is a crucial phenomenon for the overall health care delivery system. Even though a number of studies have been conducted about patient satisfaction in different health services, studies in radiology services are flimsy in Ethiopia. This study aimed at assessing patient satisfaction towards radiological service and associated factors in Hawassa University Teaching and Referral hospital.

**Methods:**

An institution based cross-sectional study was conducted among 321 adult patients presented for radiological service in the study area using stratified sampling technique. Patient satisfaction was measured using SERVQUAL (Service Quality) tool that consisted of seven items: accessibility, quality of radiological service, courtesy of radiology staff, existence of good communication with service provider and desk worker, physical environment and privacy technique. Exit interviews of patients were conducted using a structured and pretested questionnaire. Data was collected by three grade ten completed trained data collectors from May 12 to May 28, 2016. Multiple logistic regressions were used to identify independent factors associated with patient satisfaction on radiological services using SPSS version 21.

**Results:**

The overall patient satisfaction towards radiological service was 71.6%. Satisfaction to accessibility of the service was 84.5% while it was 80.6% to courtesy of the staff. Similarly, 81.6% reported satisfied with quality of the service and 59.4% and 71% of reported satisfied with physical environment and radiological service provider respectively. On the other hand, 99.7% of the respondents were dissatisfied with privacy of the service. The study revealed that patients who attended primary school (AOR = 0.317, 95% CI: 0.11–0.88), unemployed patients (AOR = 0.067, 95% CI: 0.007–0.622) and patients who had short waiting time to enter into examination room less than one hour (AOR = 4.12, 95% CI: 1.4–11.62) were factors associated with patients satisfaction.

**Conclusion:**

This study found that majority of respondents was satisfied with the radiological services. Respondent’s education level, occupation as well as duration of time taken to enter into examination room were important factors influencing the satisfaction condition. Hence, concerted effort is needed to constantly improve on patient satisfaction to better radiology returns arising from improved patient patronage. It is recommended to give great care and attention to clients during radiological examination procedure and also suggested that the department should decrease time taken to enter into examination room. On the other hand, the reasons behind more educated clients were less satisfied with radiologic service than more educated respondents need further investigation.

## Background

Quality of health services was traditionally based on professional standard practice. However, over the last decades, patient’s perception about health care has been predominantly accepted as an important indicator for measuring quality of health care and a critical component of performance improvement and clinical effectiveness [1]. It has been defined that patient satisfaction is the extent to which the patients feel that their needs and expectations are being met by the service provider [2] and also defined as the degree of congruence between a patient’s expectations of ideal care and their perception of the real care they receives [3] . The health care industry is undergoing a rapid transformation to meet the ever-increasing needs and demands of its patient population. Respect for patient’s needs and wishes, is central to any humane health care system [3, 4].

Nowadays, studying patient satisfaction becomes one of the integral components of any health care services. Patient satisfaction is a concept that has been receiving increasing attention reflecting an evolving focus in the service-oriented health care market [5]. Understanding satisfaction and associated factors have been recognized as a critical to developing many improvement strategies. Donabedian identified that the concept of patient satisfaction is complicated, irrespective of the area in which it is studied. It is a multidimensional concept; not yet tightly defined and part of an apparently yet to be determined complex model. The measurement of patient satisfaction through patient satisfaction surveys has helped organizational leaders incorporate patient perspectives as a way to create a culture where service is deemed an important strategic goal for healthcare facilities [5, 6]. However, despite their many efforts and successes with satisfaction measurement, evidence shows that more work in this area is still needed.

Patient satisfaction tended to be regarded as desirable, also critical to radiology services [7].Radiological services can be defined simply as services which are rendered to a patient visiting the radiology department which can be either routine services those carried out on a day to day basis or some special examinations that are carried out on special cases that require the use of contrast agents [8]. Despite extensive research done on defining and measuring patient satisfaction in other health departments, little attention is given to patient satisfaction with radiological services in Ethiopia. However, within the hospital system, radiological services play a major role in influencing patient satisfaction. Their high through put, diverse mix of patient populations and disease entities, procedure-related discomfort including claustrophobia, and examination types ranging from routine imaging to emergency examinations pose unique challenges [9]. In radiological services patient care which involves all the activities that are carried out before, during and after radiological diagnostic procedures to make the conditions of patient better had a great role in influencing patient satisfaction. From the practical experience it was noted, patients usually reacts to some factors that create problems in radiology department such as delay, neglect, use of harsh words on them, unnecessary repeats and preferential treatments. Patients arrived the radiology department are often worried or apparently in aggressive attitude [10].

The availability and quality of radiological service in the developing countries are generally poor [11, 12]. Ethiopia, where radiological services are not organized well, is one of the countries where overall health service has been compromised by inadequate & poorly maintained infrastructure and scarcity of health professionals [13]. Radiological service is a resource intensive unit in a hospital and most developing countries’ radiological service is expected to be poor or may not be available at all [14]. A number of studies have been conducted about patient satisfaction with other health care services in Ethiopia. Nevertheless, for different reasons including the assumption that radiological services has little or no contact with a patient, studies in radiology setting are flimsy. Therefore, as patient satisfaction with radiological service is an important issue both for evaluation and improvement of healthcare services. The present study aimed to assess patient satisfaction with radiological services and factors associated with it. As this study was the first to be conducted in Ethiopia, its result serves as indicators for radiological health service improvements to the study setting and it also becomes an initiating document for other researchers to further discuss and improve the status of radiological services delivery in Ethiopia.

## Methods

### Study setting

The study was conducted at Hawassa University Teaching and Referral Hospital from May 12 to May 28, 2016. The hospital is found in Hawassa town Sidama zone, Southern Nations Nationalities and Peoples Region(SNNPR), located 272 km South of Addis Ababa, the capital city of Ethiopia. There are two government hospitals, four private hospitals, four health centers and many private higher and medium clinics in the town. Currently, the hospital serves as a main referral center for the Southern part of Ethiopia serving about 10 million people in the region and surrounding areas [15]. The hospital, which currently has 400 beds, went operational in 2003 and is affiliated to the Hawassa University, in which the service is delivered by organizing in to four major departments: medical, surgical, pediatric, and gynecology and obstetrics departments, and other clinics like TB-HIV care unit, ART(Anti-Retroviral Therapy) clinic, Volunteer counseling and testing (VCT), ophthalmology unit, surgical and medical emergency unit, radiology unit, Intensive Care Unit (ICU) unit and Anesthesiology unit. According to the hospital annual and monthly report, the hospital has about 400 up to 500 daily outpatient visits on average [14], including approximately 70 daily radiology visits. The radiology department gives services in three main categories. Totally there are about 31 professionals in radiology department to provide radiologic services for the clients.

### Study design

Institution based cross-sectional study was conducted.

### Population

The source populations were all patients who visited and get radiological services from all outpatient and inpatient departments and also from all units in the Hospital; whereas study population were sample of selected individuals age of 18 and above, who visited and get servicesduring data collection. All patients referred from ICU unit, emergency surgical and medical unit who were in serious conditions, patients unable to communicate and patients who visited twice the service center during the data collection time for the same service were included in the study.

### Sample size determination

Sample size was calculated using single population proportion formula by considering 74.5% proportion of patient satisfaction for radiological service [8], 95% desired confidence level and 5% margin of error. After adding 10% non response rate the final sample size was 321. Stratified sampling technique was applied to draw the patients in order to get information about the radiological services in this study. First patients were stratified according to the services they visited for from the respective radiology departments; namely, conventional x-ray, ultrasound and CT-Scan services. Accordingly, during April 2016, about 1890 visited the radiology department for radiological service, in which 1200 patients visited for x-ray service, 480 patients for ultrasound service and 210 patients for CT-scan service. Then proportional sample size allocation was applied for the three services by considering annual and monthly report of patient flow in each service center of the hospital; in the same month of the preceding year (April 2015) and the month prior to the actual data collection period (April 2016). Hence, 200 samples were for x-ray service, 80 samples for ultrasound service and 41 samples for CT- scan service. Based on this on average about 70 patients visited and get radiological service daily. Finally, an exit interview was conducted on every third patient systematically selected.

### Measurement

Patient satisfaction is defined as the patients’ opinion about radiological service delivered in Hawassa University Teaching and Referral Hospital and it was measured by accessibility of service, courtesy of radiology staff, quality of radiological service, existence of good communication with service provider and desk worker, physical environment and privacy technique. To get mean score, patients response from these variables were summed up and divided by total number of questions (variables) used to measure patient satisfaction. Then it was categorized as satisfied (over all mean score of ≥3.5) and not satisfied (over all mean score of <3.5). On the other hand, patient’s attitude was measured using questions about cognitive perception of patients towards services that they get from the radiology department. Then mean score was calculated and categorized as poor attitude (over all mean score < 2.49), fair attitude (over all mean score 2.5–3.99) and good attitude (overall mean score of ≥4).

### Data collection tool and procedures

Exit interviews of patients were conducted using a structured and pretested questionnaire. Data was collected by three grade ten completed trained data collectors from May 12 to May 28, 2016

On the other hand, the questionnaire was developed after reviewing relevant literature [16, 17] on local, regional and international levels, and also in connection to Ethiopia’s social, economic and cultural background. Then the questionnaire which asked for relevant details of the radiological service system was developed. The questionnaire was divided into five parts: socio-demographic factors, patient attitude towards radiological service, patients satisfaction towards radiological service, cost and waiting time for radiological service, suggestion and comments from the patients regarding radiological service. The first part of the questionnaire was the information of the patient’s socio-demographic characteristics. The second part of the questionnaire includes six variables related to patient attitude towards radiological service with Likert Scale type of “very strongly agree scale (5), agree scale (4), uncertain scale (3), disagree scale (2), very strongly disagree scale (1)". The third part asks patient satisfaction towards radiological services with different variables related to accessibility of service, courtesy of radiology staff, quality of radiological service, existence of good communication with service provider and desk worker, physical environment and privacy technique. The fourth part of questions include 9 multiple answer questions (MAQs) about the cost and waiting time of radiological service and the last part consist of three questions about patients suggestions and comments for radiological service.

### Data quality assurance

The quality of data was controlled starting from the time of questionnaires design. First the questionnaire which was prepared in English was translated into local languages, Sidamigna and Amharic, commonly spoken in the area and it was also translated back to English to insure the consistency of the tool. Training was given for data collectors on the purpose of study and procedures of data collection for one day prior to the study. Pretest was conducted at Adare hospital out of study area in which the study population shares the same characteristics. Accordingly errors were checked, unclear and ambiguities were corrected. The data was managed through that; during data collection each subject’s questionnaire was marked with unique identification number to avoid duplication. During data collection the investigator received questionnaires from data collectors and reviewed for completeness, accuracy and consistency on daily bases. During Epi-data entry concurrent double data entry was carried out, hence it proved that there were no data entry errors. Cronbach’s Alpha was also applied in order to evaluate the internal consistency of the instrument. Cronbach‘s alpha was calculated for the 8 fields and the results were in the range from 0.57 to 0.81 and the general reliability for all items equal 0.92. This range is considered high which ensures the reliability of the questionnaire (Table 1).

**Table 1:** Cronbach’s Alpha for reliability

No	Field	No. Items	Cronbach’s Alpha
1	Patient attitude	6	0.75
2	Accessibility of radiological service	5	0.732
3	Courtesy of radiological service	5	0.6
4	Quality of radiological service	5	0.705
5	Good communication with service provider	6	0.72
6	Good communication with desk worker	5	0.81
7	Physical environment	6	0.67
8	Privacy technique	4	0.57
	Overall	42	0.92

### Analysis

Data was entered into EpiData version 3.1 and exported into SPSS for windows version 21 for analysis. Descriptive analysis was conducted to describe patients’ socio-demographic characteristics. The discrete data were described using frequencies and percentages, while the continuous variables were described using means and standard deviations. In addition, cross tabulations were done to see association on key variables to find out the factors that influenced outcome variables. Bi-variate binary logistic regression using enters method was used to get the candidate variables for multivariable logistic regression analysis in which variables with *p* < 0.25 were selected as candidate. Multivariable binary logistic regressions with odds ratios along with the 95% confidence intervals were used to identify the association between independent variables and patient satisfaction towards radiological service. A stepwise forward procedure based on the likelihood ratio was used to select the variables for the final model. Variables with more than two categories were entered in the model in the form of two “indicator” contrasts comparing each category to the last group as a reference except sex and marital status. Hosmer and Lomeshow test were used to check the goodness-of-fit of the model. The significance of each coefficient was tested by the Wald test and statistical significance was considered at *p* < 0.05. Finally, the reduced model was fitted than full model.

### Ethical considerations

Before the start of the data collection process ethical clearance letter was obtained from ethical review board of College of Health Science Jimma University and letter of cooperation was also obtained from radiology department of Hawassa University Teaching and Referral Hospital. Verbal consent was obtained from each patient by explaining that participation in the study is voluntary. Participants were explained and guaranteed for confidentiality of the information collected and also non-participation would not have negative effect on their care.

## Results

### Socio-demographic characteristics of respondents

A total of 310 adult patients participated in the study making 96.6% response rate. About 48% of the study participants were between 18 and 30 years old, males represented 51.6% of the study participants and 63.9% of the respondents were married. With respect to religion Protestants constituted 41.6%. In terms of education 37.1% and 26.1% of the participants were illiterate and had completed primary school respectively. More than one third of the respondents’ were farmers by occupation and in relation to ethnicity, 33.2% were Sidama and 28.4% were Oromo. Very small proportion 8.7% of the respondents earn above 2500 Ethiopian birr (ETB) monthly income (Table 2).

**Table 2:** Socio demographic characteristics of radiologic service users in Hawassa University Teaching and Referral Hospital, June 2016

Variable	Category	Frequency	Percent
Sex	Male	160	51.6
	Female	150	48.4
Marital status	Single	77	24.8
	Married	198	63.9
	Divorced	23	7.4
	Widowed	12	3.9
Religion	Protestant	129	41.6
	Orthodox	86	27.7
	Muslim	83	26.8
	Catholic	7	2.3
	Other	5	1.6
Age	18–30	148	47.7
	31–40	70	22.6
	41–50	51	16.5
	51+	41	13.2
Occupation	Government	47	15.2
	Farmer	107	34.5
	Merchant	48	15.5
	Housewives	28	9.0
	Student	35	11.3
	Unemployed	45	14.5
Level of education	Illiterate	115	37.1
	Primary school(1–8)	81	26.1
	Secondary school(9–12)	60	19.4
	Diploma and above	54	17.4
Ethnicity	Sidama	103	33.2
	Oromo	88	28.4
	Wolayita	40	12.9
	Gedeo	18	5.8
	Amhara	35	11.3
	Other	26	8.4
Monthly income	<600	113	36.5
	600–1200	107	34.5
	1201–2500	63	20.3
	>2500	27	8.7

### Patient’s attitude towards radiological services

This study revealed 25(8.1%) patients had poor, 184(59.4%) had fair and 101(32.6%) of patients had good attitude towards the radiological services. In assessing attitudes, patients were asked to consider friendly and courtesies’ treatment of them during examination, acceptance of their opinion by radiology staff, the way radiology staff treat them and whether the staff support them before and after exam. Hence, 217(70.0%) of them said they agreed that the radiology staff treat them in a friendly and courtesies manner, 188(60.6%) agreed that the radiology staff support them during and after exam and more than half of them also responded agree that the radiology staff accept their opinion and suggestion during the examination. Respondents also indicated that 85(27.4%) of them disagree that the radiologist/radiographer were not acting business like while performing the examination, 74 (23.9%) of them responded that the radiology staff did not greet them in friendly manner and also 63(20.3%) of them said that the radiology staff did not accept their opinion and suggestion (Table 3).

**Table 3:** Patients attitude towards radiological service of Hawassa University Teaching and Referral Hospital, June 2016

Variable	Strongly disagree	Disagree	Uncertain	Agree	Strongly agree
	*N* (%)	*N* (%)	*N* (%)	*N* (%)	*N* (%)
The radiology staff treat in a friendly and courteous manner	2(0.6)	32(10.3)	30(9.7)	217(70)	29(9.4)
The radiologist/MRT or radiographer not acting business like and impersonal	34(11.0)	85(27.4)	50(16.1)	117(37.7)	24(7.7)
Radiology staff accept your opinion	3(1.0)	63(20.3)	48(15.5)	169(54.5)	27(8.7)
The radiology staff greeted you in a friendly manner	12(3.9)	74(23.9)	35(11.3)	150(48.4)	39(12.6)
The radiology staff support you during and after examination	9(2.9)	33(10.6)	47(15.2)	188(60.6)	33(10.6)

### Patient satisfaction with radiological services

The overall proportion of patients who were satisfied with radiological services in this study was 71.6%. Out of 160 male patients, 108(67.5%) were satisfied and from 115 illiterates, 109(94.78%) were satisfied with the services. Out of 81 patients with grades 1–8, 69 (85.18%) were satisfied and also out of 69 patients with grades 9–12, 29(48.3%) were satisfied. Whereas among 54 patients who had diploma and above, 15 (27.78%) were satisfied. When considered by each service provision room, among 310 exit interviewed, 192 patients visited routine x-ray service and 147 (76.56%) were satisfied with the service. Similarly, of 81 patients visited ultrasound services, 52(64.2%) were satisfied and with regard to CT-scan service; among 37 patients visited the service 23(62.2%) were satisfied.

**Fig. 1:**
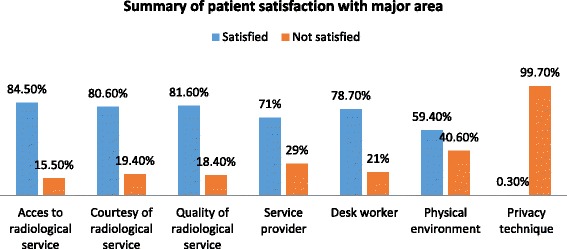
Major areas of patient satisfaction towards radiological services among radiologic visiting patients in Hawassa University referral Hospital, 2016

In assessing patients’ satisfaction with access to radiological service and courtesy of radiological service, 84.5% and 80.6% of them were satisfied with the service respectively. Patients’ satisfaction with quality of radiological service was assessed and result showed that the proportion of patients indicating that they were satisfied with quality of radiological service was 81.6%.Similarly,patient satisfaction of radiological service with service providers were assessed and its proportion was 71%.In assessing patient satisfaction of radiological service with desk workers and satisfaction with physical environment of radiological service, 78.7% and 59.4% of patients indicated that they were satisfied with service respectively. Finally, patients satisfaction with privacy of radiological service was assessed. Accordingly, the proportion of patients indicating that they were not satisfied with privacy technique of radiological service was 99.7% (Fig 1). And the overall mean score of patient satisfaction towards radiological service indicates majority 264(84.5%) were satisfied with accesiblity of the service (Table 4).

**Table 4:** Mean score of patient satisfaction towards radiological service of Hawassa University Teaching and Referral Hospital, June 2016

		Satisfaction	
Variable	Mean score	Satisfied *n* (%)	Not satisfied *n* (%)
Accessibility	3.98	262 (84.5%)	48 (15.5%)
Simplicity and trouble free of service system	3.80		
The radiology department is easy to find	4.06		
The radiology working hours are suitable	3.96		
The examination room of radiology service are convenient to ask questions	4.07		
Courtesy of radiology staff	3.82	250 (80.6%)	60 (19.4%)
Provide appropriate time for radiological examination	4.02		
Apply radiation protection technique	2.65		
The radiology staff are always willing to help patients	3.99		
The radiology staff are very polite	4.01		
Quality of radiological service	3.92	253 (81.6%)	57 (18.4%)
Competency of radiology staff to answer your question	3.47		
The instrument machine used in radiology are modern and safe	4.42		
Cleanness of examination coach sheets and pillow	3.89		
The way radiology staff keep their records is good	3.82		
Existence of good communication with service provider	3.59	220 (71%)	90 (29%)
Greet and introduce himself/ herself (Names)	3.26		
Explain the procedures of the examination	3.94		
Accept patient opinion and suggestions	4.01		
Give clear instruction during the examination and positioning	3.97		
Tell the patient right to refuse the examination	2.35		
Existence of good communication with desk worker	3.85	244 (78.7%)	66 (21.3%)
Greet and well come patients	3.29		
Register and tell appointment time(day) as soon as patient arrive	4.02		
Give information where to pay and show direction	4.04		
Give result to patient as soon as they receive it	3.94		
Physical environment	3.49	184 (59.4%)	126 (40.6%)
Waiting area has enough and comfortable sitting chairs	2.86		
Waiting area has facilities like television, air conditioners, journals, magazines or newspapers.	1.75		
The inside of radiology examination room has good ventilation	4.09		
Clear signs and directions to indicate where to go in the service area and easy to follow	4.18		
The radiology examination room is labeled with radiation warning sign	4.32		
Privacy technique	1.97	1 (0.3%)	309 (99.7%)
Availability of dressing room both for female and male	1.85		
Availability of toilets both for female and male	1.89		
Toilet is clean and suitable for use	1.93		

### Cost and waiting time for radiological service

The total money paid for radiological service was categorized into three (≤75 Ethiopian birr, 76–200 Ethiopian birr and >200 Ethiopian birr). The minimum paid money was 20 ETB which was paid for routine x-ray service, while the maximum one was 950 ETB which was paid for CT-scan service. Accordingly, 238(76.8%) patients paid less than 75 ETB, 35(11.3%) of them paid 76–200 ETB and only 37(11.9%) of patients had paid greater than 200 ETB. More than one fifth (22.3%) of patients had rated the total amount of money paid for radiological service as very cheap, 134(43.2%) of them rated as cheap, 77(24.8) of them rated as fair, 7(2.3%) as expensive and 23(7.4%) of them rated as very expensive. Respondents were asked to estimate the amount of time they spent to get radiological services to determine the total waiting time. Hence, 83(26.8%) of patients stayed less than six hours, while 42(13.5%) stayed between six and twelve hours and also two third, 185(59.7%) of them had stayed greater than twelve hours. With respect to the time taken to enter into examination room; 55(17.7%) patients waited less than 15 min, 111(35.85%) waited less than 30 min, 92(29.75%) waited between 30 min to one hour and 52(16.8%) had waited greater than two hours to enter into the examination room. With the time taken to get radiology results 29(9.4%) had reported that they got the result with less than 15 min, 55(17.7%) with less than 30 min, 41(13.2%) with less than one hour, 85(27.4%) between two to five hours, 68(21.9%) with less than one day and 32(10.3%) with greater than two days. In assessing the rate of total length of stay in radiology department; more than half of patients rated as long, nearly one fifth of them rated as very long, about 76(24.5%) as short and only 2(0.6%) of them rated as very short. About 115(37.1%) of patients reported that they were appointed to get the radiological service while 195(62.95%) of them reported they were not appointed. From 81 patients visited ultrasound service 78(91.35%) of them were appointed to get the service while from 192 patients visited routine x-ray service only 30(15.62%) of them were appointed to get the service and also from 37 patients visited CT-scan service only 7(18.9%) of them were appointed. Generally, in this way, 67.8% patients were appointed for ultrasound service, 26.1% for routine x-ray service and 6.1% were appointed for CT-scan service. On average about 33.9% were appointed for less than two weeks, 27% for less than one week, 16.5% for less than one day and 15.7% were appointed for less than three days (Table 5).

**Table 5:** Cost and waiting time for radiological service of patients in Hawassa University Teaching and Referral Hospital, June 2016

Variable	Category	Frequency	Percent (%)
Total amount of money paid for radiological services in Ethiopian birr.	≤75	238	76.8
	76–200	35	11.3
	>200	37	11.9
How do you rate the amount of money paid for radiological service?	Very cheap	69	22.3
	Cheap	134	43.2
	Fair	77	24.8
	Expensive	7	2.3
	Very expensive	23	7.4
Total length of stay in the HUTRH to get radiological service in hour	<6 h	83	26.8
	6–12 h	42	13.5
	>12 h	185	59.7
Time taken to get entered into examination room.	<15 min	55	17.7
	<30 min	111	35.8
	30 min- 1 h	92	29.7
	>2 h	52	16.8
How much time takes you to get the result/report of radiological service?	Less than 15 min	29	9.4
	Less than 30 min	55	17.7
	Less than 1 h	41	13.2
	2 h to 5 h	85	27.4
	Less than 1 day	68	21.9
	Greater than 2 day	32	10.3
How do you rate length of stay in radiology department?	Very long	60	19.4
	Long	172	55.5
	Short	76	24.5
	Very short	2	0.6
Are you appointed to get radiological services?	Yes	115	37.1
	No	195	62.9
For which service you are appointed?	Conventional x-ray	30	26.1
	Ultrasound	78	67.8
	CT-scan	7	6.1
For how many days you are appointed?	Less than1day	19	16.5
	Less than 3 days	18	15.7
	Lessthan1week	31	27
	Lessthan2weeks	39	33.9
	Lessthan3weeks	7	6.1
	Greaterthan1month	1	0.9

### Patients experience towards radiological services

Among 310 interviewed participants 292(94.2%) of them reported that they want to visit the radiology department in the future and only18 (5.8%) of them said they don’t want to visit the department in the future and also 288(92.8%) reported they recommend the radiology department service for others while about 22(7.1%) will not recommend the service. Even though the respondents were clearly explained about the significance of the research and the use of their comments or suggestion as the indicators to improve radiological service at the study area; only 210 respondents among 310 total samples interviewed gave comments or suggestions. This shows a lack of interest in giving comments regarding their personal experiences. Most of the patient expresses more than one comments or suggestion to improve radiological service (Table 6).

**Table 6:** Summary of comments and suggestions among patients of Hawassa University Teaching and Referral Hospital, June 2016

Comments and suggestions	Frequency	Percent (%)
	*N* = 265	
Long appointment times to get ultrasound service	58	21.80%
The total money paid and drugs used as contrast agent for CT-scan service too expensive	23	8.67%
Long waiting time of the x-ray and CT-scan result	54	20.37%
Long waiting time of payment for radiological services	4	1.50%
No toilet and dressing room	20	7.54%
The chairs are not comfortable and not enough chairs	72	27.16%
Bad physical environment and sanitary condition	10	3.77%
Bad reception worker behavior	1	0.37%
Radiology staff needs to improve attitude of patient service	1	0.37%
Did not know how to find where is the CT-scan room	1	0.37%
Reception workers do not give priority to elders.	19	7.16%
Bad x-ray staff attitude	1	0.37%
Bad ultrasound staff attitude	1	0.37%

### Factors associated with patients’ satisfaction towards radiological service

On binary logistic regression analyses, patients’ satisfaction towards radiological service was significantly associated with educational level, occupation, age, monthly income, marital status, patient attitudes and time taken to enter into examination room; but, on multiple logistic regression analysis it was significantly associated with educational level, occupation and time taken to enter into examination room. Accordingly, it revealed that unemployed patients were about 93.3% (AOR = 0.067, 95% CI: 0.007–0.622) less likely satisfied as compared to housewife and also the satisfaction among students were 95.8% (AOR = 0.042, 95% CI: 0.004–0.413) less likely as compared to housewife. The satisfaction among patients who attended primary school were 68.3% less likely (AOR = 0.317, 95% CI: 0.11–0.88) compared to those who were illiterates. The satisfaction among patients who had completed high school were 95% less likely (AOR = 0.051, 95% CI: 0.02–0.135) as compared to who were illiterates. Similarly, respondents who stayed shorter to enter into examination room (30 min-1 h) were 4 times (AOR = 4.12, 95% CI: 1.4–11.62) more likely satisfied as compared to those who stayed more than two hours (Table 7).

**Table 7:** Multivariate Logistic Regression analysis of patient satisfaction towards radiological service in Hawassa University Teaching and Referral Hospital, June 2016

Variable	Category	Satisfaction				
		Satisfied *n* (%)	Not satisfied *n* (%)	COR(95% CI)	AOR(95% CI)	*P*-value
Level of education	Illiterates	109(94.8)	6(5.2)	1.00	1.00	
	Primary school (1–8)	69(85.2)	12(14.8)	0.317(0.11–0.88)	0.317(0.11–0.88)**	0.028
	Secondary school (9–11)	29(48.3)	31(51.7)	0.05(0.02–0.135)	0.05(0.02–0.135)***	0.001
	Diploma and above	15(27.8)	39(72.2)	0.021(0.008–0.58)	0.021(0.008–0.58)***	0.001
Occupation	Governmental worker	21(44.7)	26(55.3)	0.03(0.004–0.23)	0.22(0.023–2.32)	0.212
	Farmer	99(92.5)	8(7.5)	0.45(0.05–3.82)	0.23(0.024–2.20)	0.202
	Merchant	36(75)	12(25)	0.11(0.014–0.90)	0.18(0.019–1.66)	0.131
	House wife	27(96.4)	1(3.6)	1.00	1.00	
	Student	13(37.1)	22(62.9)	0.02(0.003–0.18)	0.042(0.004–0.413)**	0.007
	Unemployed	26(57.8)	19(42.2)	0.05(0.006–0.40)	0.067(0.007–0.622)**	0.017
Time taken to enter exam room	≤ 15 min	37(67.3)	18(32.7)	0.99(0.44–2.24)	0.76(0.27–2.13)	0.611
	< 30 min	73(65.8)	38(34.2)	0.93(0.46–1.87)	0.84(0.34–2.08)	0.712
	30 min- 1 h	77(83.7)	15(16.3)	2.49(1.11–5.55)	4.15(1.48–11.62)**	0.007
	>2 h	35(67.3)	17(32.7)	1.00	1.00	

***p < 0.01, **p < 0.05 and * *p* < 0.1 (statistically signicant at stated *p*-value)

## Discussion

The results of this study showed that 71.6% of patients were satisfied with the radiological service they received. The result is in line with studies conducted in Nigeria, 73.4% [14], Philippines, 71% [18] and Pakistan, 71.2% [19]. Moreover, the same or comparable proportion of satisfied patients in this study may not imply that the radiological service rendered in the radiology department is of high quality and good performance. This is because patient satisfaction cannot show the real treatment outcome, which is another indicator of quality of health services [20]. But, the result of this study was lower compared to another study conducted in Nigeria [8] which may imply lower in patients’ satisfaction might be due to excess patients to the radiology department as it is the only main referral hospital used in the Southern Nation Nationalities and Peoples Region of Ethiopia including Sidama zone, Gedeo zone and also from nearby Oromia regional state of Ethiopia.

Analysis of the satisfaction condition by major areas of variables of this study indicated considerably higher satisfaction (84.5%) with the access to radiological service. About 80.6% patients reported that they were satisfied with courtesy of radiological service in this study and this is in line with the research conducted in Nigeria, 81.8% [12]; but, it is as low compared to research done in Pakistan, 87% [21]. This difference might be radiologist and radiographers were overburdened by a high patient load, administrative duties, and other commitments which may affect their courtesy. With respect to existence of good communication with service provider about 59.1% of the staff introduced themselves. This in line with the research conducted in Pakistan, 57% [21]. The similarity might be the medical ethics obligates all professions to introduce themselves before undergoing any examinations. With existence of good communication with desk worker about 78.7% patients were satisfied in this study. This is in agreement with the research conducted in Pakistan, 76.5% [18]. This similarity might be attributed to the service provided by reception worker were more or less the same. With regard to physical environment only about 59.4% respondents were satisfied in this and this was low compared to the research conducted in Nigeria, 74.8% [14]. This difference might be due to low attention given to the physical environment of health care services in Ethiopia. In this study only 0.3% of the patients were satisfied with privacy techniques of radiological services but study conducted in India Madhya Pradesh state [22] and Nepal [10] showed that about 55% and 60.6% of patient reported that they were satisfied respectively with privacy of radiological service. These variations may be due to the socio-cultural and economic status of the patients’ in the particular areas of respective countries. Besides, the source of difference may also be methodological variation. In this study more than half (59.7%) waited greater than 12 h and only about 26.8% waited less than 6 h to get the radiological service which is in contrast with study conducted in Nigeria [16] in which patient waits on average about 1 h and 14 min to access the radiological service. This large difference also might be due to excess patients to the radiology department as it is the only referral hospital used in Sidama zone, Gedeo zone and also from nearby Oromia regional state. Study conducted in Nepal [10] indicated about 80.83% and 74.9% patients had less than 30 min waiting time before and after examination respectively but in this study only 35.8% and 17.7% had less than 30 min waiting time before and after examination respectively. This difference might also be attributed to low scarcity of professional human power and equipment machine in radiology department.

This study revealed that education level was significantly associated with patient satisfaction***.*** It showed that respondents who had finished high school were 95% less likely satisfied than those who were illiterates. On the other hand, this study is in contrast with the study conducted in Nigeria [8]. The difference might be due to those educated more were more likely prone to feel small faults in the radiology department like delay, repeat and fear of radiation risk when compared to others. Again those who attended primary school were 68.3% less likely satisfied with the service as compared to those who were illiterates. This is also in agreement with the study conducted in Kuwait [23] in which patients with lower educational levels illiterate and elementary school showed a high level of satisfaction. This might be due to the educated respondents in this study need better tender care in radiological examination having being identified as the least satisfied with radiological services. In this study, unemployed patients were about 93.3% less likely satisfied when compared with housewife. This is in contrast with study conducted in Kuwait [23] which indicated unemployed respondents were more satisfied than others. This difference might be due to small number of unemployed patients were included in this study while large number of unemployed patients were included with study conducted in Kuwait.

Even though the study has much strength, it has the following limitations: First, there was lack of prior information about the prevalence of patient satisfaction towards radiological service due to unavailability of similar studies in Ethiopia before and also only few studies in other countries. Second, the content of the questionnaire included many areas with 5 items of the patients’ characteristics, 36 items of Likert scale type answer, 9 items of multiple choice questions and 1 items of multiple answer question and the multiple choice questions included 3 to 5 answer options. Hence the questionnaire was quite long and patients should have to spend more than 25 min to finish the questionnaires and some people might feel impatient, which could affect the quality of data. Thirdly, the patient’s diagnosis, symptoms or illness severity as factors predicting satisfaction, which may affect their satisfaction was not included in this study.

## Conclusion

Studying patient satisfaction with radiological service is very important given the fact that patients are usually unfamiliar with the complexities of radiological examination and the sophistication of equipment involved in the procedure. In this study, it was found that the majority of the respondents were satisfied with the radiological services. Patients were satisfied with access to radiological service, courtesy of radiological service, quality of radiological service, also with service provider and also with desk workers; but, more than half and almost all patients were dissatisfied with the physical environment and privacy techniques of the radiological service respectively. Respondents level of education, occupational status and time taken to enter into examination room were found to influence patient satisfaction towards radiological service. Hence, concerted effort is needed to constantly improve on patient satisfaction to better radiology returns arising from improved patient patronage. Greater attention needs to be given to the interplay between patients’ socio-demographic factors and time taken to enter into examination room. Based on our finding provision of great care and attention during service provision, decreasing time taken to enter into examination room, reducing appointment time for ultrasound and decreasing CT scan payment are recommend. Furthermore, as this study was the first with patient satisfaction towards radiological service, periodical study focusing on patients’ satisfaction in the department and hospital should be implemented to keep up with the change of the phenomena.

## Abbreviations

ART: Anti-Retroviral Therapy
CT-scan: Computerized tomography scan
DCBE: Double Contrast Barium Enema
HUTRH: Hawassa University Teaching and Referral Hospital
MAQs: Multiple answer questions
MDGs: Millennium development goals
PSQ: Patient satisfaction questions
SERVQUAL: Service quality
SNNPR: Southern Nations Nationalities and Peoples Region
SPSS: Statistical package for social science
WHO: World Health Organization
